# Case of Cervical Tumor and Operation

**Published:** 1848-05

**Authors:** H. Ganson

**Affiliations:** Batavia, N. Y.


ART. IV.—*Case of Cervical Tumor and Operation.* By H. GANSON, M. D.

At the request of a medical friend, I submit for publication in the pages of your valuable journal, a brief notice of a cervical tumor, and a short account of the operation of its extirpation, which was performed upon the person of John McGregor, in the month of December last, in the town of LeRoy, in this state.

At the time of the operation, the diseased mass had had nearly four years growth, was nodulated to the feel, and was of a cartilaginous consistence. Its situation was upon the right side of the neck, and occupied more than two thirds of the whole cervical region.

During its development, it had deeply penetrated and insinuated itself amongst all the important organs in the neck, carrying in its course the base of the tongue nearly to the roof of the mouth, and crowding the whole floor of the pharynx far backward. The process of deglutition, and articulation, had become quite impracticable. Two large lobes of the tumor firmly embraced the sub-maxillary bone on this side.

Before beginning the incision, it was suggested by Dr. Walter Cary, of Buffalo, who was, by my particular request present, and from whom I received very valuable assistance during the various stages of dissection, that the jaw-bone itself might be involved. Accordingly, after having first secured the common carotid at a short space above the clavicle, at which point it became necessary to tie this vessel, from the magnitude of the tumor, an elliptical incision was freely made, extending from the lobe of the ear to the mesiac line before the chin. After having continued the dissection down to the sub-maxillary bone, a large part of this structure was found to be extensively necrosed. The diseased portion of the jaw-bone was carefully separated by the metacarpal and Hayes' saw. This being accomplished, the operation was continued until the whole mass was completely removed.

The glosso-pharyngeal nerve and the digastricus muscles, were completely enveloped by the tumor. But they were not injured during the operation.

The whole operation required one hour and forty minutes, and the diseased mass weighed sixteen ounces.

Besides Dr. Cary, there were present, Drs. Wm. Sheldon, Barret, Chamberlin, Burwell, and the two Drs. Meecham, also Mr. Green, at that time a student of mine, to all of whom I am much indebted for their kind assistance during this trying operation. The patient is a man about 45 years of age, and inhaled, with satisfactory results, the sulphuric ether. The wound healed kindly, and he has recovered from the effects of the operation.

BATAVIA, N. Y., January, 1848.

